# Successful Immobilization of Lanthanides Doped TiO_2_ on Inert Foam for Repeatable Hydrogen Generation from Aqueous Ammonia

**DOI:** 10.3390/ma13051254

**Published:** 2020-03-10

**Authors:** Miroslava Edelmannová, Martin Reli, Lenka Matějová, Ivana Troppová, Lada Dubnová, Libor Čapek, Dana Dvoranová, Piotr Kuśtrowski, Kamila Kočí

**Affiliations:** 1 Institute of Environmental Technology, VŠB-Technical University of Ostrava, 17. listopadu 15, 708 00 Ostrava-Poruba, Czech Republic; miroslava.edelmannova@vsb.cz (M.E.); lenka.matejova@vsb.cz (L.M.); ivana.troppova@vsb.cz (I.T.); kamila.koci@vsb.cz (K.K.); 2 Faculty of Chemical Technology, University of Pardubice, 573 Studentská, Pardubice, Czech Republic; lada.dubnova@student.upce.cz (L.D.); libor.capek@upce.cz (L.Č.); 3 Institute of Physical Chemistry and Chemical Physics, Faculty of Chemical and Food Technology, Slovak University of Technology in Bratislava, Radlinského 9, SK-812 37 Bratislava, Slovakia; dana.dvoranova@stuba.sk; 4 Faculty of Chemistry, Jagiellonian University, Gronostajowa 2, 30-387 Kraków, Poland; Piotr.kustrowski@uj.edu.pl

**Keywords:** ammonia, hydrogen production, immobilized and powder photocatalyst, lanthanides, TiO_2_

## Abstract

We describe the successful possibility of the immobilization of a photocatalyst on foam, which is beneficial from a practical point of view. An immobilized photocatalyst is possible for use in a continuous experiment and can be easily separated from the reactor after the reaction concludes. Parent TiO_2_, La/TiO_2_, and Nd/TiO_2_ photocatalysts (containing 0.1 wt.% of lanthanide) were prepared by the sol-gel method and immobilized on Al_2_O_3_/SiO_2_ foam (VUKOPOR A) by the dip-coating method. The photocatalysts were investigated for the photocatalytic hydrogen generation from an aqueous ammonia solution under UVA light (365 nm). The evolution of hydrogen was compared with photolysis, which was limited to zero. The higher hydrogen generation was observed in the presence of 0.1 wt.% La/TiO_2_ than in 0.1 wt.% Nd/TiO_2_. This is, besides other things, related to the higher level of the conduction band, which was observed for 0.1 wt.% La/TiO_2_. The higher conduction band’s position is more effective for hydrogen production from ammonia decomposition.

## 1. Introduction

Nowadays, renewable energy sources and the elimination of harmful substances from the environment belong to the most frequently researched and desired areas of interest, especially if the elimination of pollutants can lead to a renewable energy source. For example, hydrogen is a very promising future environmentally friendly energy carrier. Its combustion is free of pollutant emissions. H_2_ can be relatively easily stored and transported in the form of hydrogen containing compounds including ammonia [1].

Ammonia is widely used as a chemical material in many production processes. However, it is also a pollutant with strong negative effects on human health and nature. The ammonia removal from contaminated water is not an easy task [2,3,4]. The most promising way to achieve this goal is its photocatalytic decomposition, which enables ammonia removal and the production of renewable energy sources in the form of hydrogen (Equation (1)). The energy required for this reaction may be supplied directly from solar energy, if a proper photocatalyst is designed and used [5,6].

(1) $$2\;NH_{3}\;\overset{hv,\;\;photocatalyst}{\to }\;N_{2}+\;3H_{2}$$

The photocatalytic decomposition of ammonia usually proceeds at room temperature and atmospheric pressure. Ammonia shows many advantages as the hydrogen source: (a) it is a carbon-free system; therefore CO_2_ is not formed during the decomposition; (b) it is effortless to store and transport; and (c) the content of H_2_ in one molecule (NH_3_: 17.6%) is relatively high when compared to other hydrogen storage carriers [5,7].

Titanium dioxide is considered as one of the most suitable photocatalysts for the environmental applications due to its high photocatalytic activity, biological and chemical inertness, strong oxidizing power, long-term stability against photocorrosion as well as self-cleaning and antibacterial effects [8]. However, the practical application of TiO_2_ is limited due to the high recombination rate of photogenerated electron-hole pairs and its wide bandgap, which requires UV light irradiation for activating the photocatalytic reaction. For that reason, extending the light absorption range of TiO_2_ into visible light range is highly desirable. This can be achieved, among other things, by metal doping of TiO_2_ [9] or by the preparation of binary composites, for example, with graphene oxides [10]. Many different elements have been introduced into TiO_2_ over the past decades, whereas lanthanide doped TiO_2_ has attracted interest as it, for example, could form complexes with various Lewis bases [11,12].

Thus far, the vast majority of photocatalytic reactions use photocatalysts in the powder form. However, powder represents additional challenges, especially in photocatalytic water treatment, namely during the complete separation of powder photocatalysts from water by filtration. Therefore, fixing the photocatalytically active components in powder form to a support material, termed “immobilization”, with high surface area can be beneficial for photoactivity and stability. Properly immobilized photocatalytic materials allow the use of the liquid stream and the gas stream, because there is no entrainment of particles of a nanometer size. Well immobilized photocatalysts will suffer less physical damage over time due to environmental conditions and can be used repeatedly. The photocatalytic activity depends on the used support material and immobilization method (e.g., sol-gel, solvent deposition, electrophoresis, thermal spraying as well as chemical and physical vapor deposition). A suitable immobilization does not cause a reduction in photocatalytic activity and reduction of specific surface area; in addition, photocatalyst particles with good adhesion to the selected support material should be used. The reduction of a specific surface area would have a negative impact on the ability to remove contaminants. Another essential aspect of the immobilization process is the final crystalline structure of the photocatalyst on a carrier [13,14,15,16].

For this reason, in this work, we investigated parent TiO_2_ and lanthanum or neodymium doped TiO_2_ photocatalysts (with 0.1 wt. % of lanthanide) that were immobilized on inert Al_2_O_3_/SiO_2_ foam. The photocatalytic performance of the obtained materials was examined in the photocatalytic decomposition of ammonia for H_2_ generation in a homemade photoreactor. The main goal of this work was the immobilization of TiO_2_ based photocatalysts on foam for the repeatable generation of hydrogen from aqueous ammonia.

## 2. Materials and Methods

### 2.1. Preparation of Photocatalysts

Photocatalysts were synthesized by using the reverse micelles-based sol-gel method. The pure TiO_2_ micellar solution was prepared following the procedure described in [17], mixing 40.6 mL of cyclohexane, 18 mL of non-ionic surfactant Triton X-114, 0.610 mL of distilled water, and 10 mL of titanium (IV) isopropoxide as a Ti source. The micellar solutions for the preparation of TiO_2_ photocatalysts containing 0.1 wt.% of lanthanum or neodymium where prepared, according to [18,19] with using lanthanum(III) nitrate hexahydrate (La(NO_3_)_3_·6H_2_O, Aldrich) and neodymium (III) nitrate hexahydrate (Nd(NO_3_)_3_·6H_2_O, Aldrich) as metal cation sources. First, the proper amount of lanthanum (0.00718 g) or neodymium (0.00704 g) precursor was dissolved in 3 mL of absolute ethanol. Second, this La or Nd-containing ethanol solution was added to the mixture of cyclohexane, Triton X-114, and distilled water and stirred for 20 min. Finally, the proper amount of titanium (IV) isopropoxide was added and the final solution was mixed for a further 20 min. All prepared solutions were left to stand overnight in closed bottles. After that, they were used for photocatalyst deposition on the Al_2_O_3_/SiO_2_ foam.

The TiO_2_, La/TiO_2_, and Nd/TiO_2_ coated foams were prepared using the dip-coating method. The prepared solutions were dip-coated using COATER5 (idLAB) on the substrate (Al_2_O_3_/SiO_2_ foam, VUKOPOR A, Lanik Ltd., Boskovice, Czech Republic, measured S_BET_ = 0.7 m^2^/g). For detailed information about the substrate, see the Supplementary Materials (Figure S1). The coating parameters were: dipping speed of 150 mm/min, delay of 60 s, and drawing speed of 60 mm/min. The coated foams were left to dry at ambient temperature for 4 h and then calcined at 450 °C for 2 h with a heating rate 3 °C/min. The process of dip-coating and calcination was repeated three times.

### 2.2. Characterization Methods of Photocatalysts and Photocatalytic Tests

The textural, structural, optical, and electronic properties of the studied photocatalysts were characterized by nitrogen physisorption [19,20], powder x-ray diffraction (XRD, Rigaku SmartLab diffractometer, Rigaku, Tokyo, Japan) [19,20], x-ray fluorescence (XRF, Elva X energy-dispersive X-ray fluorescence spectrometer, Elvatech Ltd., Kiev, Ukraine) [20], diffuse reflectance UV–Vis spectroscopy (DRS UV–Vis, GBS CINTRA 303 spectrometer, GBC Scientific Equipment, Braeside, Australia) [20,21], x-ray photoelectron spectroscopy (XPS, VG SCIENTA R3000, Prevac Ltd., Rogów, Poland), Raman spectroscopy with a difference 532 nm laser source was used, electron paramagnetic resonance (EPR, EMXplus, Bruker, Bruker Daltonics Ltd., Billerica, Massachusetts, USA), and photoelectrochemical measurements [19]. More details are given in the mentioned references. For microscopic investigations of the photocatalysts, a scanning electron microscope (SEM: FEI Quanta 450, Waltham, Massachusetts, USA) with a field emission gun (FEG) was used.

Ammonia decomposition was carried out in a homemade batch reactor with and without a photocatalyst (photocatalysis and photolysis, respectively) using UVA irradiation. The irradiation source was placed in a horizontal position. The reactor was filled with 100 mL of ammonium hydroxide solution (0.883 g/l ammonia), sealed, and purged with helium for 30 min before the start of the reaction. The gaseous samples were discontinuously taken and analyzed for 0–6 h by a gas chromatograph equipped with a barrier discharge ionization detector (BID). The reproducibility tests and blank tests were performed. For a detailed photo of the reactor, see the Supplementary Materials (Figure S2).

## 3. Results and Discussion

### 3.1. Structural and Textural Properties of Photocatalysts in Its Powder Form

The basic properties of the investigated photocatalysts in the powder form are summarized in Table 1. The x-ray fluorescence measurements confirmed the amounts of lanthanum and neodymium were very close to the intended 0.1 wt.%. The amount of metal in the photocatalysts was measured by x-ray fluorescence (XRF) analysis. Concentration of La in the La/TiO_2_ photocatalyst was determined to be 0.08 wt. % by using a wave-dispersion x-ray fluorescence spectrometer (Spektroskop MAKC-GV, Spectron NPO Ltd., St. Petersburg, Russia). The concentration of Nd in the Nd–TiO_2_ photocatalyst was determined to be 0.1 wt. % by using Elva X energy-dispersive x-ray fluorescence spectrometer (Elvatech Ltd., Kiev, Ukraine). The resulting value was determined as the average of three measurements [20,22].

**Figure 1:**
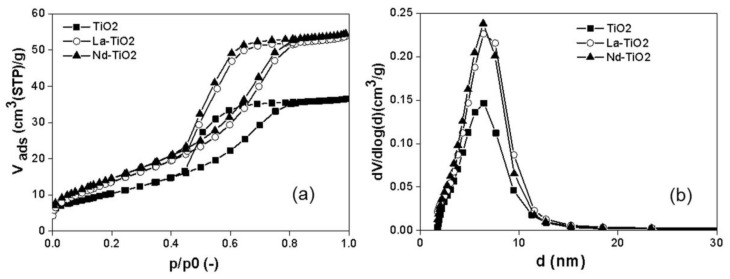
(**a**) Nitrogen adsorption–desorption isotherms and (**b**) evaluated pore-size distributions of the investigated photocatalysts.

The nitrogen adsorption–desorption isotherms of the materials showed a similar shape (IV type of the IUPAC classification, Figure 1a), which corresponded to the mesoporous character of all investigated materials with negligible participation of microporosity. The nitrogen physisorption showed an increasing specific surface area with lanthanum or neodymium loading in the photocatalyst (Table 1). The pore size distribution also (Figure 1b) confirmed the trend of increasing specific surface area. The increased specific surface area after the addition of lanthanum was in agreement with the literature [23,24]. The increased specific surface area was explained by Liu et al., who claims that La_2_O_3_ easily occupy defect sites on the surface of anatase particles and prevents the transformation of anatase to rutile and increases the grains.

**Figure 2:**
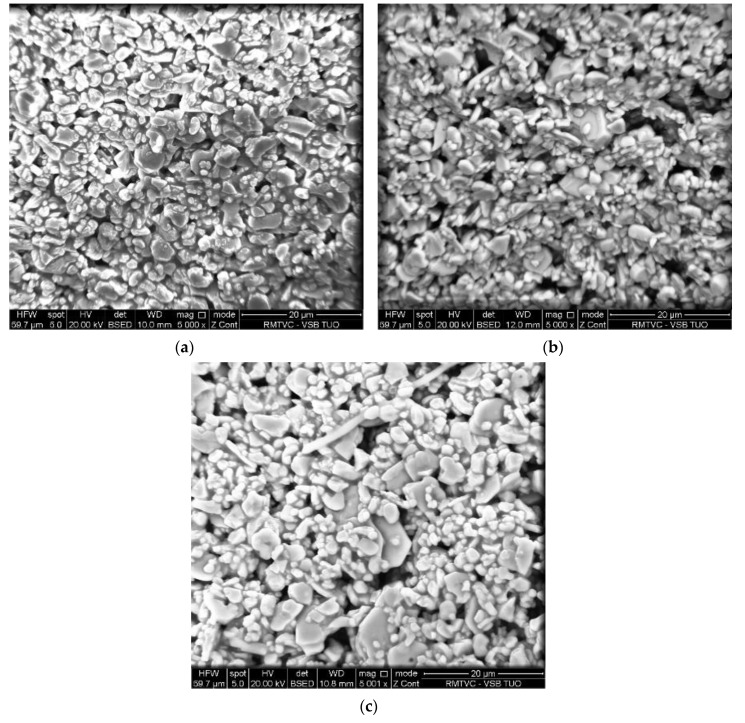
Scanning electron microscopy images of (**a**) TiO_2_, (**b**) La/TiO_2_, and (**c**) Nd/TiO_2_.

The morphology of the photocatalysts immobilized on the Al_2_O_3_ foam was examined by SEM analysis (Figure 2). Figure 2a shows an image of pure TiO_2_ immobilized on Al_2_O_3_ foam and Figure 2b,c show images of immobilized TiO_2_ doped by 0.1 wt.% of lanthanum and neodymium, respectively. It is clear that all samples had a very similar morphology and formed irregular agglomerates with a hint of layered structure. An EDS analysis was also performed; however, it was impossible to confirm the presence of Ti, Nd, or La in the chemical composition since the base materials were in the significant majority and only peaks of the foam material were detected.

**Figure 3:**
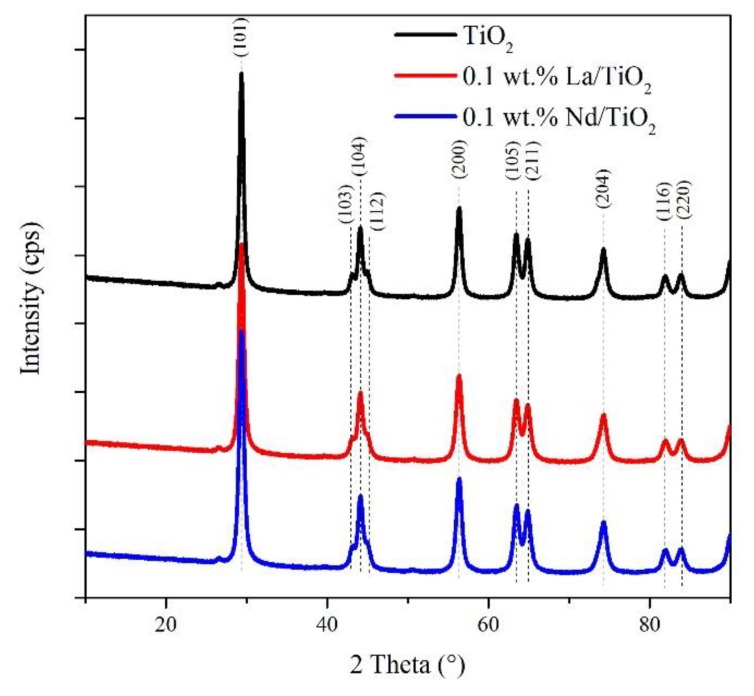
X-ray diffraction patterns of pure TiO_2_, La^3+^ and Nd^3+^ ions doped TiO_2_.

The XRD patterns of pure TiO_2_, La/TiO_2_, and Nd/TiO_2_ are shown in Figure 3. All diffraction lines were assigned to the presence of the tetragonal modification of TiO_2_ anatase and corresponded to characteristic planes by the International center for diffraction data (ICDD) PDF card no. 00-021-1272 (space group I4_1_/amd and lattice constants *a,b* = 3.7852 Å and *c* = 9.5139 Å). The detected anatase reflections were marked with appropriate (h, k, l) symbols. An additional polymorphic form of TiO_2_ as well as Nd or La related phases (oxide forms) as impurities were not identified. This indicates a high dispersion of a small amount of La/Nd oxide. The refined lattice parameters, cell volumes, and crystallite sizes are shown in Table 2. It is clear that the addition of lanthanides reduced the crystallite size of the final photocatalyst, suggesting that lanthanides prevent agglomeration. Similar results have been previously reported [23].

The addition of lanthanides did not significantly change the band gap energy value of the prepared photocatalysts compared to the pure TiO_2_. This is in agreement with our previous results observed for La/TiO_2_ and Nd/TiO_2_ materials containing 0.2–1.5 wt.% of lanthanide [18]. In principle, another reason for changing the value of the band gap energy could be the presence of different phases of titanium dioxide. However, the preparation of these materials always led to the presence of the pure anatase form in TiO_2_, La/TiO_2_, and Nd/TiO_2_ (Figure 3) [18,20].

The XPS measurements revealed the presence of Ti, O, and contaminating C on the surface of the studied materials. Only unmeasurable traces of lanthanides were identified in the case of the La- and Nd-doped TiO_2_ samples. In all photocatalysts, two photoelectron peaks at 458.4 eV and 464.1 ± 0.1 eV, corresponding to Ti 2p_3/2_ and Ti 2p_1/2_ levels, confirmed the existence of Ti^4+^ exclusively [18,19]. On the other hand, surface oxygen was distributed in the form of lattice O^2−^ (529.7 eV) as well as OH^−^ species (530.8 ± 0.1 eV). Doping of TiO_2_ with the low amount of La and Nd (0.1 wt. %) did not affect the surface concentration of oxygen (lattice and chemisorbed oxygen or/and hydroxyl species) determined by XPS (Table 1). The complete XPS results are shown in the Supplementary Materials (Table S1).

Raman spectroscopy confirmed the exclusive presence of the anatase phase (Figure S3). The addition of La had a marginal influence on the position of the most intensive band at 143 cm^−1^, whereas the introduction of Nd to TiO_2_ caused a slight red shift (0.5 cm^−1^). The shift of the band maximum is related to both the anatase crystal size and the amount of oxygen vacancies [25,26]. In the case of Nd/TiO_2_, the observed red shift could be connected to a lower crystallite size of Nd/TiO_2_ in contrast to TiO_2_ and La/TiO_2_.

### 3.2. Photocatalytic Hydrogen Production from Ammonia

**Figure 4:**
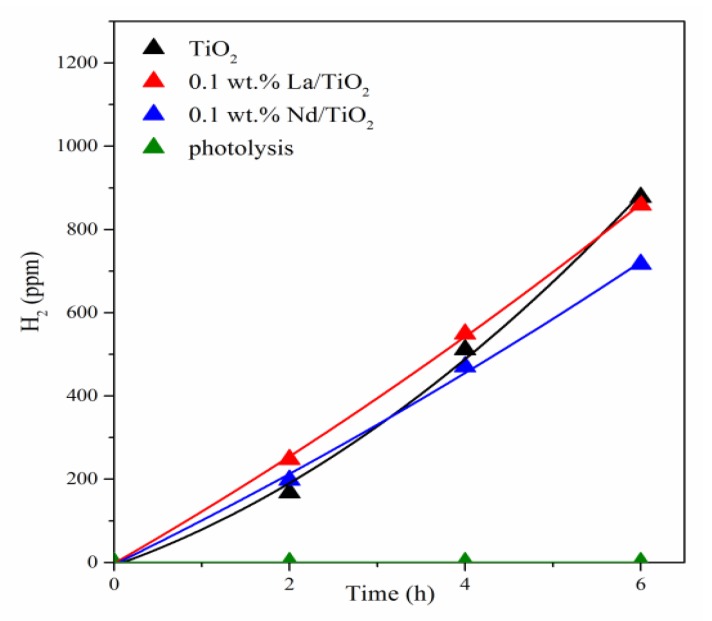
H_2_ yields vs. reaction time during photolysis and in the presence of the investigated photocatalysts immobilized on foam.

The hydrogen production from the photocatalytic decomposition of NH_4_OH is shown in Figure 4. All prepared photocatalysts produced significantly higher amounts of hydrogen than in the case of photolysis. The 0.1 wt.% La/TiO_2_ possessed a slightly higher yield of hydrogen than the 0.1 wt.% Nd/TiO_2_. For TiO_2_, the yield of hydrogen was similar to 0.1 wt.% Nd/TiO_2_ at the beginning of the reaction and was slightly higher after 4 h. All catalytic tests were measured repeatedly.

Due to the fact that no possible by-products of ammonia decomposition (NO_3_^−^, NO_2_^−^, and N_2_O) were observed, the ammonia decomposition can be described by Equation (1), involving the formation of nitrogen and hydrogen exclusively. Furthermore, the photochemical and photocatalytic decomposition of ammonia is well fitted by the first order rate law model [21]. Conversion of ammonia (Equation (3)) was derived from hydrogen mass balance (Equation (2)).

(2) $$n_{H_{2}}=n_{H_{2}}^{0}+\frac{3}{2}n_{NH_{3}}^{0}\cdot X_{NH_{3}}$$

(3) $$X_{NH_{3}}=\frac{\frac{2}{3}\left(n_{H_{2}}-n_{H_{2}}^{0}\right)}{n_{NH_{3}}^{0}}$$

where $n_{NH_{3}}^{0}$ is the number of moles of NH_3_ at the beginning of the reaction (*t* = 0); $n_{H_{2}}^{0},\;n_{H_{2}}$ are the numbers of moles of H_2_ at the beginning of the reaction (*t* = 0) and at different times during the photocatalytic reaction, respectively; and $X_{NH_{3}}$ is the conversion of NH_3_.

An integral form of material balance for a batch photoreactor with ideal mixing was used for data processing:

(4) $$ln\frac{1}{1-X_{NH3}}=k\cdot t$$

where *k* is the kinetic constant (h^−1^) and *t* is the reaction time (h). The kinetic data were evaluated according to the first order rate law. Kinetic constant for the photocatalysts immobilized on foam was slightly higher for La/TiO_2_ than for Nd/TiO_2_ (in agreement with Figure 4). The kinetic constant value for TiO_2_ was between the values for lanthanide-doped TiO_2_ photocatalysts. However, its value was affected by the highest experimental error (Table 3).

The photocatalytic decomposition of ammonia is a complex reaction, which occurs through a series of redox steps and number of intermediates including various radical species. Generally, the effectiveness of photocatalytic reactions depends on the textural, structural, and optical characteristics of the photocatalyst such as the specific surface area, band gap energy, particle size, recombination rate of electrons and holes, and so on. In this work, we focused on using the photocatalysts immobilized on foam. We used a low lanthanide loading on TiO_2_ (ca 0.1 wt. %). Such a low lanthanide content led to the approximately same concentration of surface oxygen species (Table 1), band gap energy value (Table 1), and the presence of the pure anatase phase (Figure 3, Figure S3). In contrast to TiO_2_, lanthanide doped TiO_2_ photocatalysts only possessed a slightly higher specific surface area (Table 1) and a slightly lower TiO_2_ crystallite size (Table 2).

For decomposition of ammonia, the energy of electron formation in the conduction band must be higher than the N_2_/NH_4_OH reduction potential, which is −0.77 V versus standard hydrogen electrode (SHE) [21]. The pure TiO_2_ had a wide band gap (*E*_g_ = 3.0 eV) and the position of the valence band was at 2.14 V, which was determined from the XPS measurement. The calculated conduction band potential of −0.86 V is sufficient for the photocatalytic decomposition of ammonia. In the case of the 0.1 wt.% La/TiO_2_ photocatalyst, the position of valence band was the same and the band gap energy was also the same (*E*_g_ = 3.0 eV). Therefore, the position of the conduction band was the same (−0.86 V) as in the case of pure TiO_2_. In the case of 0.1 wt.% Nd/TiO_2_, the situation was somewhat different. Considering the fact that the band gap was the same as in previous cases (*Eg* = 3.0 eV) and the position of valence band was lower, at about 70 mV, the energy of the electron formation on the conduction band (−0.79 eV) was completely on the edge of the reduction potential of ammonia. For the 0.1 wt.% Nd/TiO_2_ photocatalyst, the carriers had the most unfavorable potentials for the ammonia decomposition. This could be the reason for the slightly lower value of produced hydrogen among the studied photocatalysts.

NH_3_ and NH_4_^+^ species should be considered as candidates for reactions with charge carriers and reactive oxygen species in the photocatalytic ammonia decomposition over undoped/doped titanium dioxide. The aminyl (azanyl •NH_2_) and hydroxyl (•OH) radicals are formed through the interaction of photogenerated holes with adsorbed NH_3_ and HO^−^/H_2_O, respectively, in aqueous media upon UVA exposure. Furthermore, the production of hydrogen radicals by the reduction of NH_4_^+^ with photogenerated electrons may be expected [27]. Thus, the EPR spin-trapping technique was applied for the detection of non-persistent radicals using 5,5-dimethyl-1-pyrroline N-oxide (DMPO) as the spin trapping agent [28]. Upon the UVA exposure (λ_max_ = 365 nm, 18 mW cm^−2^) of undoped/doped titania in anoxic aqueous suspensions containing 8 mM ammonia and DMPO, in all reaction systems, only the ^•^DMPO–OH spin adduct (spin-Hamiltonian parameters, *a*_N_ = 1.504 mT, *a*_H_ = 1.474 mT, *g* = 2.0057) was monitored at low concentrations. The EPR signals corresponding to ^•^DMPO–NH_2_ or ^•^DMPO–H spin-adducts were not detected. We suppose that under the given experimental conditions, their reactions with DMPO are not favorable, and those radicals are involved in a variety of reactions [27]. Despite the fact that the spin trapping technique failed in the detection of ^•^NH_2_ and ^•^H intermediates, it can be helpful in testing the photocatalytic activity of undoped and doped titania following the generation of ^•^DMPO–OH in the aerated aqueous suspensions [28].

**Figure 5:**
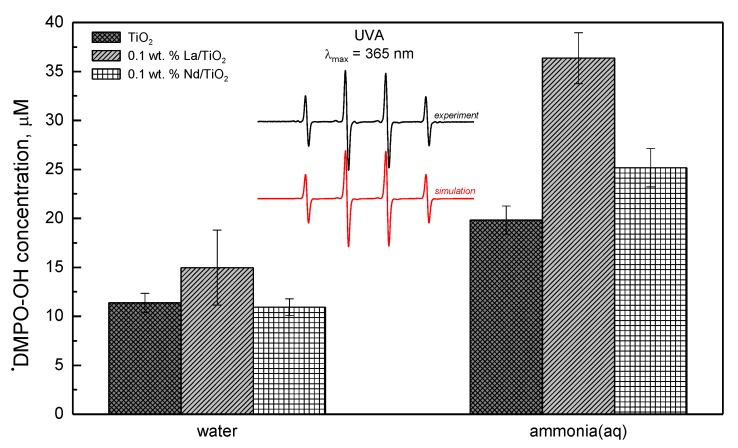
The concentration of the ^•^DMPO–OH spin-adduct evaluated from the EPR spectra obtained upon 330 s UVA photoexcitation (λ_max_ = 365 nm; irradiance, 18 mW cm^−2^) of aerated aqueous suspensions of pristine and doped TiO_2_ without/with ammonia in the presence of the DMPO spin trap. Inset: The experimental and simulated EPR spectra (magnetic field sweep width 8 mT) of ^•^DMPO-OH obtained upon UVA irradiation of TiO_2_/DMPO/air. (c(TiO_2_) = 2.00 g L^−1^; c_0_(DMPO) = 0.04 M, c_0_(NH_3_) = 0.008 M).

Figure 5 represents the concentration of the ^•^DMPO–OH spin-adduct evaluated from the integral intensity of the EPR signal upon 330 s UVA exposure (radiation dose 5.9 J cm^−2^) in the ammonia-free aqueous titania suspensions, in comparison with the data obtained in the presence of ammonia. Comparable concentrations of ^•^DMPO–OH were found using pristine and doped TiO_2_ photocatalysts in ammonia-free systems, evidencing only a limited effect of doping. Theoretically, the photocatalytic oxidation of NH_3_ in TiO_2_ suspensions in the presence of oxygen includes the formation of various intermediates, which may react with ^•^OH in the complex competitive reactions and such a process reflects in the decrease in the ^•^DMPO-OH concentration. However, in our experiments, the presence of ammonia led to the detection of higher concentration of ^•^DMPO–OH for all photocatalysts (Figure 5). Considering the complex character of the photocatalytic system containing DMPO, this increase can be caused by: (i) influence of ammonia on DMPO adsorption; (ii) stabilization of ^•^DMPO–OH spin-adduct in an alkaline environment; and (iii) increased formation of ^•^OH via photolysis/decomposition of photogenerated H_2_O_2_ or nitrite [29,30]. Moreover, significantly higher ^•^DMPO–OH concentrations were detected for the doped TiO_2_ photocatalysts, mainly for the 0.1 wt.% La/TiO_2_ photocatalysts.

In order to bring the photocatalysis closer to industrial use, a suitable design of a photocatalytic reactor and photocatalyst form should be developed. The design of a photocatalytic reactor is efficient only if there is effective interaction between three phases (liquid, solid, and light) for liquid systems. The distributions of the photocatalyst and light inside the photoreactor are important to increase the conversion and yield rates [31]. From this point of view, the production of hydrogen over photocatalysts immobilized on foam has a strong advantage and allows for the recycling of the photocatalyst by its simple separation/removal from the liquid phase [13].

## 4. Conclusions

The pure TiO_2_ and lanthanides (0.1 wt. % La or Nd) doped TiO_2_ were prepared by the sol-gel method and immobilized on foam and investigated as photocatalysts in the generation of hydrogen from an aqueous ammonia solution. Low lanthanide doped TiO_2_ photocatalysts (ca 0.1 wt. % of lanthanide) led to the approximately same concentration of surface oxygen species, band gap energy value, and presence of pure anatase phase. In contrast to TiO_2_, lanthanide doped TiO_2_ photocatalysts possessed higher specific surface area and lower TiO_2_ crystallite size. La/TiO_2_ possessed a higher production of hydrogen than Nd/TiO_2_. The higher level of the conduction band, which was observed for 0.1 wt.% La/TiO_2_, was more effective for the ammonia decomposition. This fact contributes to the higher hydrogen generation in the presence of 0.1 wt.% La/TiO_2_ than in Nd/TiO_2_. TiO_2_ immobilized on foam represents an attractive possibility for its continuous utilization as it does not require separation from the liquid phase.

## Figures and Tables

**Table 1:** Textural, structural, and optical properties of the investigated photocatalysts (in powder form).

Photocatalyst	S_BET_^1^	V_tot_^2^	Amount of Metal La/Nd Determined by XRF	Indirect Band Gap	Lattice and Chemisorbed Oxygen or/and Hydroxyl Species
		(cm^3^(STP)/g)	(wt.%)	(eV)	(at.%)
**TiO_2_**	39	37	-	3.0	63.4
**0.1 wt.% La/TiO_2_**	53	54	0.08	3.0	63.7
**0.1 wt.% Nd/TiO_2_**	56	55	0.10	3.0	63.6

^1^ The specific surface area, S_BET_, was calculated according to the classical Brunauer–Emmett–Teller (BET) theory for the p/p_0_ = 0.05–0.25 of the nitrogen adsorption–desorption isotherm. ^2^ The total pore volume, V_tot_, was determined from the nitrogen adsorption isotherm at maximum p/p_0_ ~0.9900.

**Table 2:** Structural properties of the La/Nd/TiO_2_ photocatalysts.

Photocatalyst	Crystallite Size (nm)	Lattice Parameters		Cell Volume (nm^3^)
		*a*(Å)	*c*(Å)	
**TiO_2_**	**14.0**	3.7853	9.51301	13.630
**0.1 wt.% La/TiO_2_**	12.1	3.7844	9.50750	13.617
**0.1 wt.% Nd/TiO_2_**	8.8	3.7880	9.51650	13.655

**Table 3:** Kinetic constants with statistical errors for the prepared photocatalysts.

Photocatalyst	Kinetic Constant for Photocatalysts Immobilized on Foam (10^4^ h^−1^)
**TiO_2_**	2.55 ± 0.15
**0.1 wt.% La/TiO_2_**	2.68 ± 0.02
**0.1 wt.% Nd/TiO_2_**	2.33 ± 0.04

## Supplementary Materials

The following are available online at https://www.mdpi.com/1996-1944/13/5/1254/s1, Figure S1: Photo of the Vukopor^®^ A. Figure S2: Picture of reactor for photocatalytic study on decomposition of ammonia over a photocatalyst immobilized on foam. Figure S3: Raman spectra of TiO_2_, La/TiO_2_, and Nd/TiO_2_ photocatalysts. Table S1: Surface concentration of Ti and O elements determined by XPS.
