# Supplementary material for: Successful Immobilization of Lanthanides Doped TiO2 on Inert Foam for Repeatable Hydrogen Generation from Aqueous Ammonia
Source: Materials (Basel). 2020 Mar 10;13(5):1254. doi: 10.3390/ma13051254 (PMC7085088; doi:10.3390/ma13051254)
Supplement: Supplementary file 1 [file materials-13-01254-s001.pdf]

*materials*

*Article*

# Successful Immobilization of Lanthanides Doped $TiO_2$ on Inert Foam for Repeatable Hydrogen Generation from Aqueous Ammonia

**Miroslava Edelmannová [1], Martin Reli[1,*], Lenka Matějová [1], Ivana Troppová [1], Lada Dubnová [2], Libor Čapek [2], Dana Dvoranová [3], Piotr Kuśtrowski [4] and Kamila Kočí [1]**

[1] Institute of Environmental Technology, VŠB-Technical University of Ostrava, 17. listopadu 15, 708 00 Ostrava-Poruba, Czech Republic; miroslava.edelmannova@vsb.cz (M.E.); lenka.matejova@vsb.cz (L.M.); ivana.troppova@vsb.cz (I.T.); kamila.koci@vsb.cz (K.K.)
[2] Faculty of Chemical Technology, University of Pardubice, Studentská 573, Pardubice, Czech Republic; lada.dubnova@student.upce.cz (L.D.); libor.capek@upce.cz (L.Č.)
[3] Institute of Physical Chemistry and Chemical Physics, Faculty of Chemical and Food Technology, Slovak University of Technology in Bratislava, Radlinského 9, SK-812 37 Bratislava, Slovak Republic; dana.dvoranova@stuba.sk
[4] Faculty of Chemistry, Jagiellonian University, Gronostajowa 2, 30-387 Kraków, Poland; Piotr.kustrowski@uj.edu.pl
* Correspondence: martin.reli@vsb.cz; Tel.: +42-0597-327-304

Received: 17 January 2020; Accepted: 6 March 2020; Published: date

## Supplementary Materials

### *2.1. Preparation of Photocatalysts*

VUKOPOR is the $Al_2O_3$ foam (Figure S1) with following parameters: specific surface area 0.6 $cm^2/g$, apparent density of ceramic body 2.35 $g/cm^3$, and porosity of ceramic body 33%. The utilized foam had a height of 1 cm and a diameter of 7 cm.

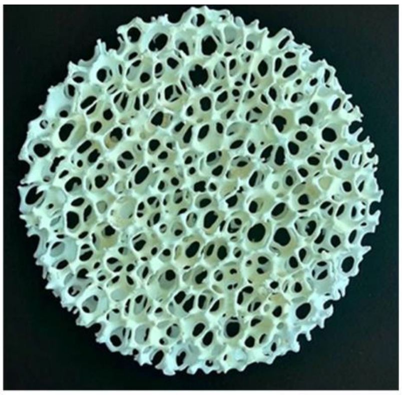

**Figure S1.** Photo of the Vukopor® A.

### *2.2 Photocatalytic Tests*

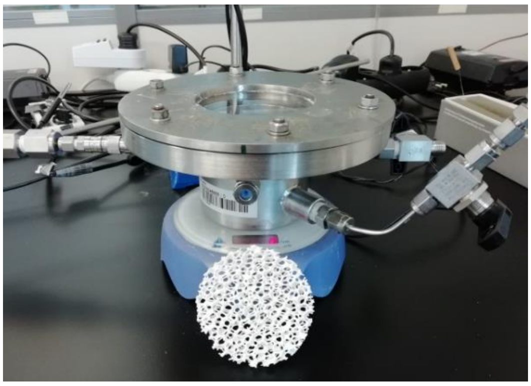

**Figure S2.** Picture of the reactor for photocatalytic study on the decomposition of ammonia over a photocatalyst immobilized on foam.

## 3. Results and Discussion

### *3.1. Structural and Textural Properties of Photocatalysts in Its Powder Form*

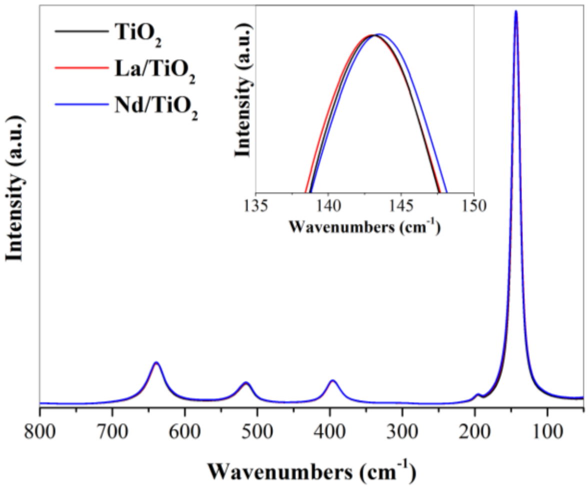


**Figure S3.** Raman spectra of $TiO_2$, La/$TiO_2$, and Nd/$TiO_2$ photocatalysts.

**Table S1.** Surface concentration of Ti and O elements determined by XPS.

| Photocatalyst | $Ti^{4+}$ in $TiO_2$ (at.%) | Oxygen Lattice $O^{2-}$ (at.%) | Oxygen $OH^-$ (at.%) | Total O (at.%) | Carbon (at.%) |
|---|---|---|---|---|---|
| $TiO_2$ | 27.4 | 54.77 | 8.61 | 63.4 | 9.2 |
| 0.1 wt.% La/$TiO_2$ | 27.8 | 57.63 | 6.10 | 63.7 | 8.4 |
| 0.1 wt.% Nd/$TiO_2$ | 27.6 | 57.02 | 6.56 | 63.6 | 8.8 |

© 2020 by the authors. Submitted for possible open access publication under the terms and conditions of the Creative Commons Attribution (CC BY) license (http://creativecommons.org/licenses/by/4.0/).
